# Classification of incidental findings in polytrauma computed tomography

**DOI:** 10.1186/s13244-026-02229-0

**Published:** 2026-03-02

**Authors:** Daniela Kildal, Rainer Braunschweig, Stefan Reske, Nadine Egenrieder, Daniel Vogele, Meinrad Beer

**Affiliations:** 1 Diagnostic and Interventional Radiology, University Hospital Ulm, Ulm, Germany; 2 Hôpital du Valais, Upper Valais Hospital Center SZO, Radiology, Brig, Switzerland; 3 Department of Radiology, University Hospitals Erlangen, Erlangen, Germany; 4 HBK Heinrich-Braun-Klinikum, Universität Leipzig + Jena, Zwickau, Germany

**Keywords:** Whole body imaing, Multiple trauma, Tomography, X-ray computed, Incidental findings

## Abstract

**Objectives:**

Whole-body computed tomography (WBCT) is the standard procedure for examining severely injured patients. In addition to trauma-caused pathologies, a high number of non-trauma-related pathologies, incidental findings (IFs) are found regularly but often underestimated in WBCT. A standardized image analysis and a classification of IFs regarding their clinical graduation is of paramount interest for both treatment concepts and outcomes. The present study is aimed at developing and validating a feasible classification system. We evaluated WBCT scans regarding IFs and classified the IFs into 5 degrees of severity.

**Materials and methods:**

The present retrospective study included 1475 polytrauma patients from two maximum care hospitals who underwent a WBCT scan. Medical reports and CT scans were then reviewed for IFs.

**Results:**

The 83.8% of patients had suffered trauma-related injuries, and in 83.9%, IFs were found. The patients’ age and gender significantly influenced the number and severity of IFs. Older and female patients tended to have more IFs. IFs are not described more often in patients without trauma-related findings (15%) than in patients with traumatic injuries (6%). Based on an analysis of 476 literature sources, we classified 511 different IFs into 5 categories. Most of them fell into categories 1 (variant) and 2 (benign), but 24% fell into categories 3 (follow-up), 4 (needs clarification), and 5 (needs treatment), requiring monitoring, clarification, or immediate treatment.

**Conclusion:**

Due to the high rate of IFs, standardized image analysis and IFs classification are of utmost importance for both the patients’ further treatment and the healthcare system’s refinancing of resources.

**Critical relevance statement:**

IFs in polytrauma CT scans are common and, if not adequately addressed, can negatively impact patient outcomes—therefore, this 5-level classification standardizes interpretation and provides specific recommendations for further action, evaluation, or treatment.

**Key Points:**

- In polytrauma, IFs are as common as trauma-related findings.
- There is a need for standardized classification.
- For IFs in WBCT scans, we propose a classification into 5 categories, labeled as IF-RADS 1–5.

**Graphical Abstract:**

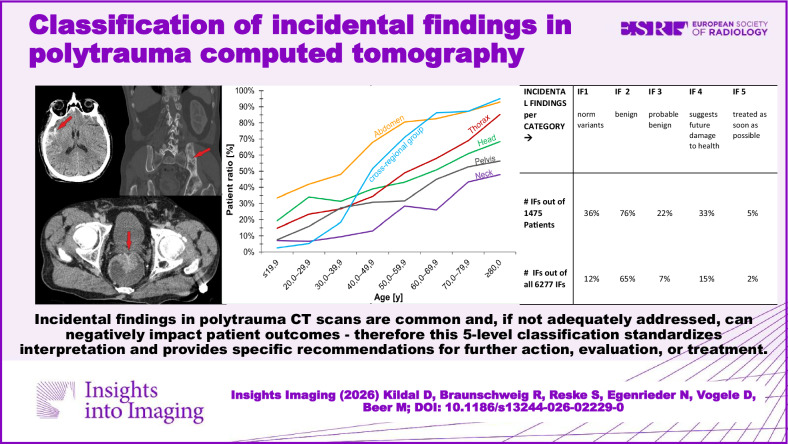

## Introduction

The widespread and still increasing use of whole-body computer tomography (WBCT or “full-body CT” or “pan-scan”, including head, C-spine, chest, abdomen, and pelvis) goes along with a rising detection rate of IFs, defined as (additional) findings which are not related to the primary clinical indication for the imaging examination [1–4]. Sometimes, these IFs may have an even greater impact on the health of the trauma patient than the injuries requiring a WBCT [3] itself.

There is only a small amount of information on the frequency and the medical significance of IFs in imaging diagnostics [5]. Depending on imaging modality, body region, and the patients’ age, data on IF-frequency in imaging research studies vary significantly [3, 6, 7]. Moreover, there are no rules that doctors should inform patients, colleagues, and clinicians dealing with the follow-up treatments of the IFs [8]. Finally, the disclosure of IFs to research patients and research volunteers is of ethical relevance, too [9]. Schlett et al noted that IFs can be of great importance to the patients on one side (illness can be detected and treated early), and can represent even a psychological burden on the other side (for example, if IFs, which are not yet clinically relevant, are made known to the patient and the patient focuses on the finding and gets worried). Therefore, recommendations for dealing with IFs would be helpful in the clinical context, as well as for optimized, workflow-oriented tools (such as structured reporting and follow-up performances) [10].

The inclusion of IFs in diagnostic imaging may necessitate further investigations [7, 11]. There is no doubt that unnecessary tests must be avoided because of their potential harmfulness to patients and related costs [1]. On the other hand, untreated IFs may entail serious consequences for the patient and even higher costs due to the progression of pathology. So, the period of hospitalization was noted to be longer for patients with IFs compared to those without [12]. A simple classification of IFs regarding their clinical relevance could lead to cost reduction and less harmful treatment by avoiding unnecessary diagnostics.

The classification of IFs in WBCT into clinically relevant risk categories may enable the identification of individuals at higher risk. In order to quickly distinguish important, time-critical IFs from non-critical or even clinically unimportant ones, a simple classification into severity levels would be useful. Previous studies on IFs suggested between 2 and 4 categories, in some cases without clinical recommendation [3, 4, 11, 13–16]. To date, there have not been any general recommendations for dealing with incidental findings (IFs) [17, 18].

Therefore, we evaluated WBCT scans with regard to non-trauma-related pathologies and classified them into five categories. We opted for five categories with ascending pathology because existing and widely established classifications (TI-RADS, LI-RADS, PI-RADS, etc.) are based on five categories as well and have been widely accepted. Furthermore, these classifications include recommendations for the treatment of single organs, which is helpful for adequate written reports by radiologists.

## Materials and methods

This study was conducted in accordance with the Declaration of Helsinki and approved by the Ethics Committee of the University of Ulm (AZ: 302/17), and written informed consent was waived with approval. This research paper was not funded by any public, commercial, or non-profit agency.

This retrospective pilot study was based on an RIS/PACS query. Patients were identified by a RIS-based, full-text search for CT images, and in addition, the patients’ history was checked. Following statistical consultation, a sample of 1500 polytrauma patients with whole-body CT scans (out of a total of 5629 patients, over a 7-year observation period) was randomly selected for review. Twenty-five WBCTs had been performed at external clinics and had to be excluded, because no written report was available. The study is therefore based on a detailed analysis of 1475 cases.

All WBCTs were first interpreted clinically by an on-call radiologist and were secondly reviewed by a senior radiologist as part of routine care. For the present study, both the medical reports and the corresponding WBCT image data were retrospectively reviewed by members of the working group (consisting of doctoral students and attending physicians from the Radiology Department) specifically for non-traumatic IFs. In cases of uncertainty, consensus was sought with a senior consultant or the working group leader. IFs from other imaging examinations were not considered, nor were iatrogenic changes, medical materials, or older trauma results investigated. Trauma-related findings cannot be considered in this paper due to space constraints and methodological reasons.

The IFs were assigned to one of 6 anatomical regions: head, neck, thorax, abdomen, pelvis, and a cross-regional group. The patients were divided into 8 age groups (≤ 20, 20.0–29.9, 30.0–39.9, 40.0–49.0, 50.0–59.9, 60.0–69.9, 70.0–79.9, and ≥ 80 years of age).

Recommendations from specialized groups, such as the Fleischner Society or the white papers of the American College of Radiology (ACR) IFs Committee, and well-known classifications, such as the BI-RADS classification, CT/MRI Liver Imaging Reporting and Data System (LI-RADS), the Bosniak classification of cystic renal masses, or the modified Breast Imaging Reporting and Data System. [19], were included, wherever applicable.

**Fig. 1:**
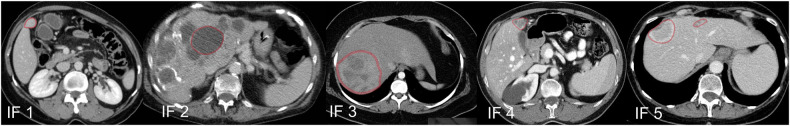
Example of IF 1–5 in the liver. IF1 is a benign variant, IF2 cysts, IF3 benign but requiring monitoring—in this case, a large adenoma, IF4 a finding requiring further investigation, and IF5 a tumorous mass

**Fig. 2:**
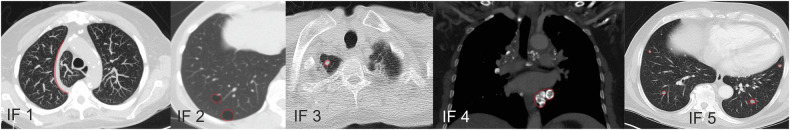
Example of IF 1–5 in the lung. IF1 is a variant, IF2 are single emphysematous bulla, IF3 is a pulmonary nodule of 6 mm and therefore requires monitoring, IF4 is a finding requiring further investigation—here, silicosis or sarcoidosis is suspected, and IF5 is lung metastases

**Fig. 3:**
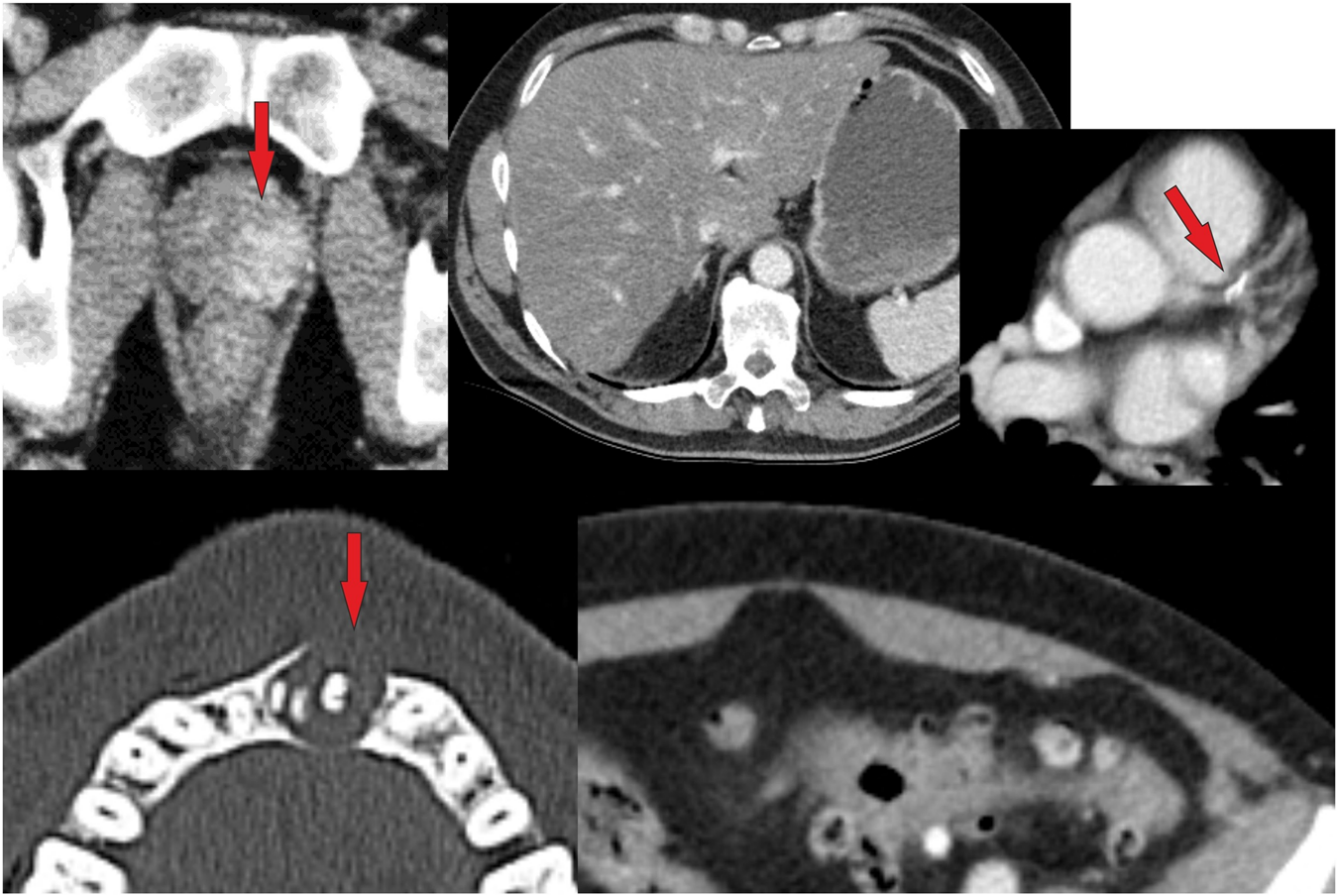
A male polytrauma patient (fall from > 3 m under the influence of alcohol) was found unconscious. The WBCT scan revealed the following findings: frontal soft tissue hematoma, subdural hematoma, sternum fracture, multiple rib fractures, scapula fracture, radius and ulna fracture. In addition to the injuries mentioned above, the following IFs were noted (see above): hyperdense prostate lesion, a large mass in the left mandible, fatty liver, arteriosclerosis (coronary artery disease, aortic sclerosis), diverticulosis, and advanced degenerative changes. According to Table 1, the hyperdense lesion in the prostate was classified as IF 5 (suspected carcinoma, which was proven histologically later). The lytic mandibular mass was classified as IF 4 (suspected ameloblastoma, elective investigation recommended) (Bone Rads 4, aggressive imaging features)

**Fig. 4:**
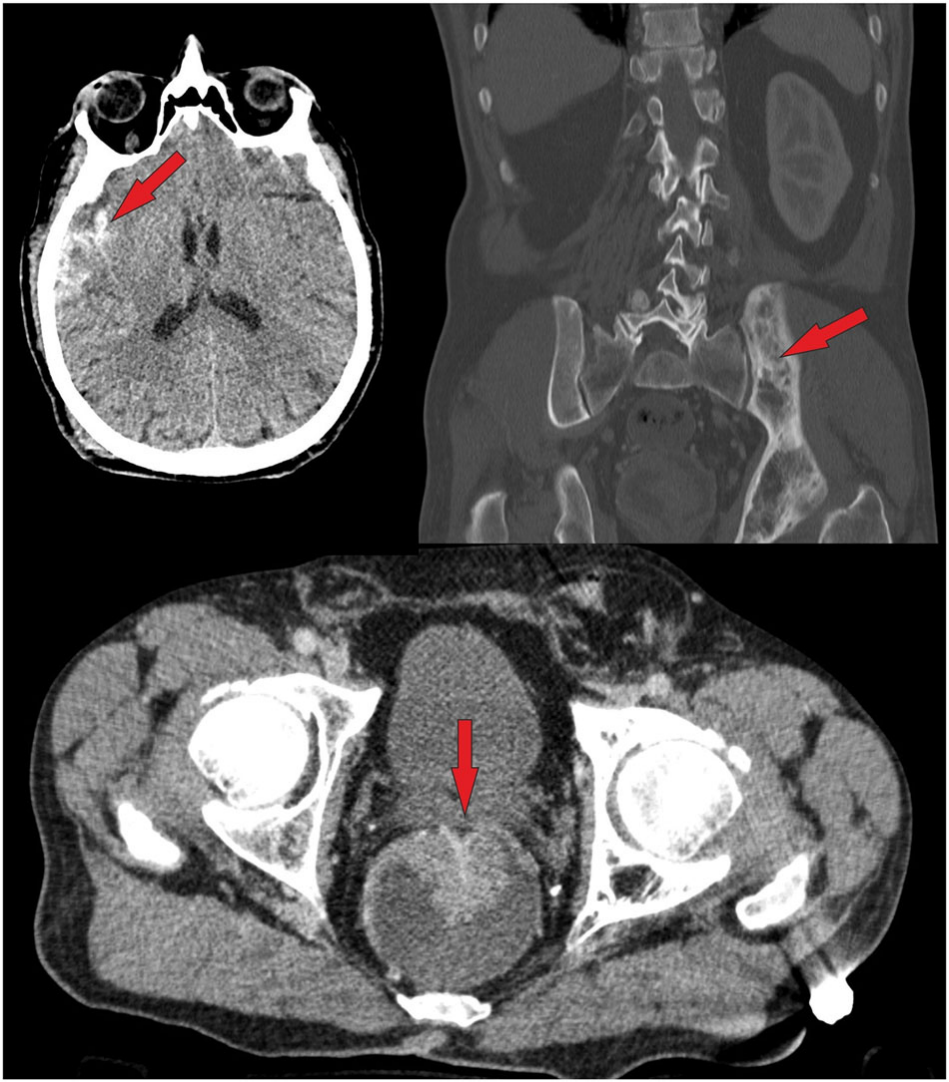
Patient with intracranial bleeding and some fractures of the extremities (shown in additional X-rays). IFs on whole-body CT scan: left pelvic fibrous dysplasia (IF 2, bone rads 1—benign lesion without aggressive features), and a suspicious mass in the rectum, classified (acc. to Table 1) as IF 5 because it was interpreted as a carcinoma

The resulting incidental finding classification includes five incidental finding categories (IFCs) named as IF-RADS (Incidental Findings-Reporting and Data System) categories presented in Table 1. Examples for IFC 4 and IFC 5 are shown in Figs. 1–4.

**Table 1:** Classification of IFs^a^ into 5 IFCs, respectively, 5 IF-RADS categories

IFC	Description	Examples	Recommendation
1	Anatomical norm variants	Lobus venae azygos, accessory spleen, liver cyst, or hemangioma	Documentation in the medical report.
2	Benign IF, without immediate consequences	Pulmonary bullae, emphysema, degenerative changes, cysts with small calcifications	Documentation in the medical report.
3	Probable benign IF	Solitary pulmonary nodule between 6 and 8 mm. Renal cysts with smooth, minimally thickened, enhancing walls	Documentation in the assessment with recommendations for follow-up.
4	IF that suggests future damage to health	Round lung lesions > 8 mm, suspected sarcoidosis, renal cysts with one or more enhancing walls or septa ≥ 4 mm	Documentation in the assessment with recommendations for elective clarification.
5	IF that should be evaluated or treated as soon as possible	Acute pneumonia, cholecystitis, ileus, embolism, suspected carcinoma, metastasis	Documentation in the assessment with reference to urgent clarification and/or therapy.

^a^ 476 literature sources were used for analysis and classification. An APP is planned to present a complete list of 511 IFs

Based on a population of 8.25 billion, the representative sample size of 1475 subjects results in a confidence level of 98% and a margin of error of 3%. All data analyses were performed using Microsoft® Excel and SPSS® Statistics Version 24.0.

## Results

One thousand four hundred seventy-five patients were included. The mean age of all patients was 46.4 years (from 0.7 years to 101 years, median: 45 years). Most of the patients (68%) were male. The prevailing cause of accidents was traffic accidents (50.2%), followed by falls (30%), sports accidents (5%), and others (4%). For 9% of the patients, no information about the cause of the accident could be found. One thousand two hundred thirty-six patients (83.8%) had a CT-detectable injury, whereas in 239 patients (16.2%) no traumatic injury could be detected. Traumatic injuries are evaluated in other studies and are not pursued further in this paper due to space constraints.

A total of 6277 IFs were recorded. IFs were identified in most of the patients (83.9%, *n* = 1237). In 1041 patients (84.2% of patients with IF), more than one IF was detected. In 111 cases, IFs were not described primarily, but seen in the second review (8%), more often in patients without trauma related findings (15%) than in patients with traumatic injuries (6%). Five hundred eleven different IFs were found. Among other things, we found normal variants (IF1) such as accessory spleens in 143 patients (9.6%), azygos lobes in 14 patients (0.9%), and a lusoric artery in 8 patients (0.5%). A significant difference was found between male and female patients with regard to the number of IFs (*p* < 0.001). The median of IFs was 4.0 in female patients and 3.0 in males.

**Fig. 5:**
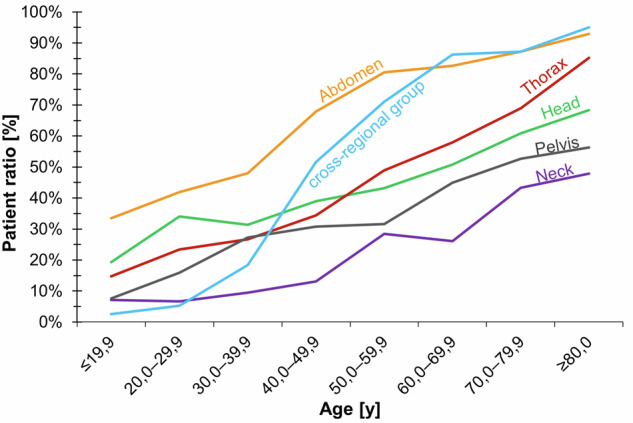
Number of IFs as to gender and age

IFs were most common in the abdomen (34.8%), thorax (17.8%), head (14.0%), pelvis (10.5%), and the neck (5.9%). A large proportion of IFs (16.8%) were localized across regions (e. g., degenerative/arthritic changes in several regions) (for further details see Fig. 5).

**Fig. 6:**
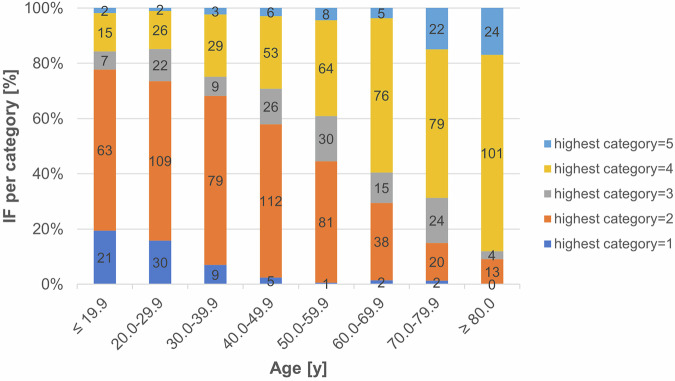
Relation between patients’ age and the severity of IFs (note: categories as defined in Table 1

The age of patients with IFs was significantly higher than that of patients without IFs (49.8 years and 22.5 years, respectively, (*p* = *p* < 0.001)). There was a significant positive correlation between patient age and the severity level of IFs (*p* < 0.001) (see Figs. 5 and 6 for more details).

A total of 72 patients (4.9%) had category 5 IFs, which required immediate clarification or treatment. Approximately half of the IFs in this category (45%, *n* = 46) were pathologies with a high degree of suspicion of malignancy, 20% (*n* = 20) were acute inflammatory processes, such as pneumonia, that required treatment, and serious vascular findings, such as thrombosis, pulmonary artery embolism, or aneurysms, amounted to 13% of IFs. The remaining 23% IFs in category 5 included acute clinical conditions such as acute urinary obstruction, ileus, incarcerated inguinal hernias, and others. Table 2 gives a survey of IFs per category.

**Table 2:** IFs in IF-RADS category 1–5 (cave—since an average of several IF tests were recorded per patient, the total percentage may exceed 100%) and in % of all IFs

IFs per CATEGORY →	IF1 norm variants	IF 2 benign	IF 3 probable benign	IF 4 suggests future damage to health	IF 5 should be evaluated or treated as soon as possible
# IFs out of 1475 patients	36%	76%	22%	33%	5%
# IFs out of all 6277 IFs	12%	65%	7%	15%	2%

## Discussion

In the present study, we identified a higher percentage of IFs (83.9%), compared to previous studies (26% to 76%) [2, 8, 20, 21]. With 5.1 IFs, the average number of IFs per patient was higher than in other studies (between 1.3 and 3.1) [2, 4, 8, 14, 21]. The discrepancy in the results can be partly attributed to different inclusion criteria for IFs in the different studies. Our study showed an approximately equal share of trauma-related and IFs. This is in accord with other studies, which found even more IFs than traumatic injuries [1].

The rate of clinically relevant IFs was found to be 5% in this study. This is below those observed and reported by Grattan et al (11%) [8], close to those found by Kolbeinsson et al (6,6%) [15], but much higher than those reported by James et al (1%) [2]. Possible explanations for that could be the following: there is no general classification for IFs, previous studies on IFs suggested between 2 and 4 categories [2–4, 11, 13, 14, 16], and there have not been any general recommendations for dealing with IFs so far [17, 18]. Therefore, the comparability of these studies is severely limited.

In other studies, the absence of an IF classification is associated with a lack of systematic documentation and missing communication of IFs to the doctors for further treatment or to the patients directly [2, 4, 8, 14, 16]. Structured reporting improves the documentation and detection of IFs. The probability of guideline-consistent treatment for IFs is significantly higher for CT scans with standardized reporting than for scans without it [22]. To improve the detection of IFs, we recommend structured reports: each body-region (skull, vertebral column, chest, abdomen, extremities) has to be analyzed in tissue, lung, and bone window settings as usual. For the appraisal of WBCT scans of polytrauma-patients a systematic reading by at least two radiologists should be performed. This is desirable for both trauma-related and IFs. Therefore, using a checklist could be helpful.

Moreover, for standardized image analysis and reporting, standardized CT protocols are of paramount interest. For that particular reason, we recommend the Guidelines for Polytrauma-CT by the European-Radiological Society [23], the Guidelines for Polytrauma by the AWMF (working group of the medical scientific societies) [24], and Quality-Assurance-Guide-Lines for computed tomography [25], because of their actual alignment to each other.

As a matter of fact, most IFs are not clinically significant or do not require further treatment [2]. There is a risk that people with (harmless) IFs will be frightened or have an impact on their current quality of life [26, 27]. The participants in Oerlemans et al [27] were primarily affected by uncertainty. A cascade of further diagnostics or follow-up checks will not give further benefit to the patients concerned. Moreover, this may lead to healthy people being exposed to unnecessary risks, unwarranted harm, and increased costs [9, 26, 27]. On the other hand, for some IFs, an early detection may be crucial for the patient’s health, such as the early detection of a tumor. Furthermore, the study by Oerlemans et al showed that some participants, knowing about their IF, became more aware of their lifestyle and quit smoking, for example [27].

Therefore, in radiological reports, IFs must be clearly emphasized and distinguished from trauma findings. Studies have shown that follow-up care for IFs is often inadequate, and IFs are not mentioned in discharge summaries in approximately half of the cases. Institutions can benefit from developing guidelines for reporting and follow-up treatment of IFs in trauma patients [28]. A classification of IFs is therefore important to highlight critical findings while avoiding unnecessary diagnostics and overtreatment.

To some extent, the results of the present study are limited. On the one hand, this is due to its retrospective character. On the other hand, despite classifying 511 different IFs in 5 categories, we assume that some minor findings were omitted. Considering the analysis of 476 literature sources, one cannot keep up with the latest developments without electronic support. We are about to specify the details for imaging analysis and detailed scoring of IFs and develop an easily accessible App-based solution for such a classification.

The documentation of IFs and their clinical impact was found to be incomplete in other studies [4]. Therefore, we emphasize the necessity of a second assessment of CT images focusing on IFs either by the responsible radiologist as a second reading or by a second radiologist (see above).

Similar to previous studies and compared to cadaver studies, we received rather low values, among other findings, for the prevalence of accessory spleen (according to our data, 9.6% vs literature data 10–30%, azygos lobe 0.9% vs 1%, and lusoric artery 0.5% vs 0.5–2.5%) [29–32]. We achieved a population-representative sample size of 1475 with a confidence level of 98% and a margin of error of ±3.03%. This provides a high degree of reliability for our results, allowing us to draw meaningful conclusions about the study population. The IFs reported here suggest that our study also slightly underestimates the number of IFs. In particular, the large difference in non-diagnosed IFs between traumatized and non-injured patients is remarkable. This is not a satisfaction of search error as we first assumed, because we found not-described IFs more often in patients without trauma-related findings (15%) than in patients with traumatic injuries (6%). This should be a topic for future research. In an initial survey of the examiners, the use of text modules in patients with no traumatic findings in WBCT may be one of the reasons. Other colleagues generally reported no “benign” or “age-appropriate” secondary findings. This would underline the importance of a classification system since some radiologists apparently do not understand the significance of IFs. Overlooking/failing to report clinically insignificant IFs may, in other cases, be related to the emergency situation, as the focus is clearly on assessing serious/life-threatening injuries.

Our main concern for a clinically helpful classification of IFs is to integrate widely known and accepted classifications, such as BI-RADS (breast), C-RADS (CT Colonography), LI-RADS (Liver), Lu-RADS (Lung), TI-RADS (Thyroid), PI-RADS (Prostate), O-RADS (ovarian-adnexal), or Bone-Rads, which record many findings in a standardized way already [33].

We classified IFs into five categories of increasing severity, each of which also includes recommendations for further interventions. Accordingly, we propose “IF-RADS” as the relevant classification of IFs. This should help to compare IFs in further studies and to delineate them for clinical purposes.

## Conclusion

IFs in (poly-) traumatic patients are very common. Their incidence is the same as for trauma-related findings. According to our and other international studies, 5%–10% of all IFs have a life-relevant impact for patients.

A standardized image analysis and clinical graduation (classification), as well as some management recommendations, e.g., how to deal with IFs as far as the patients’ interests are concerned, are missing and would be very helpful. In addition, from an economic perspective, patients with clinically relevant IFs should receive treatment at an early stage. Highlighting and classifying IFs in radiological reports and providing recommendations for further diagnostics and treatment are clearly advantageous for physicians and patients, and can be implemented without additional costs.

Our retrospective study presents an IF classification system consisting of five IF-RADS categories with ascending degrees of severity, which gives treatment recommendations for each category as the main clinical benefit for the patients. The positive side effect is the use of the existing data for better clinical effectiveness.

The long-term perspective could be, firstly, a speech recognition or App-based system to incorporate IFs into the daily routine of radiological reports. Secondly, in cases of unclear injuries/illnesses or so-called “long-lie trauma” without polytrauma, a whole-body CT scan should be considered, as a high number of IFs can be expected, especially in older patients.

## Data Availability

Data supporting the reported results can be found in the hospital’s PACS system.

## Abbreviations

ACR: American College of Radiology
IFC: Incidental finding categories
IF-RADS: Incidental findings-reporting and data system
IFs: Incidental findings
WBCT: Whole-body computed tomography
